# Bleeding Pseudocyst of the Pancreatic Head. The role of Omentoplasty and Local Hemostasis

**DOI:** 10.1155/1994/23181

**Published:** 1994

**Authors:** Francesco Stipa, Adolfo Gavelli, Claude Huguet

**Affiliations:** 1 Department of Surgery Princess Grace Hospital Principality of Monaco France; 2 Department of Surgery Centre Hospitalier Princesse Grace Avenue Pasteur Monaco, Principauté de Monaco 98000 France

## Abstract

Treatment of bleeding psedoaneurysms and pseudocysts of the pancreas is controversial. Surgical treatment with pancreatic resection or trancystic arterial ligation is not always satisfactory since postoperative mortality rate is high, especially for lesions located in the pancreatic head and rebleeding is not unusual. Two patients with bleeding pseudoaneurysms (one post traumatic, one spontaneous) and one with a hemorrhagic pseudocyst of the pancreatic head were treated surgically with arterial suture and omentoplasty. Bleeding was controlled in all, without any postoperative mortality or morbidity. No rebleeding occurred with a follow up of 33, 26 and 12 months. Trancystic ligation of bleeding vessels with omentoplasty may be a useful approach, which should be compared to arterial embolization in the future.


*HPB Surgery*, 1994, Vol. 8, pp. 123–128
Reprints available directly from the publisher
Photocopying permitted by license only

© 1994 Harwood Academic Publishers GmbH
Printed in Malaysia

### Bleeding Pseudocyst of the Pancreatic Head. The role of Omentoplasty and Local Hemostasis

FRANCESCO STIPA, ADOLFO GAVELLI and CLAUDE HUGUET

Department of Surgery, Princess Grace Hospital, Principality of Monaco

Treatment of bleeding psedoaneurysms and pseudocysts of the pancreas is controversial. Surgical treatment with pancreatic resection or trancystic arterial ligation is not always satisfactory since postoperative mortality rate is high, especially for lesions located in the pancreatic head and rebleeding is not unusual. Two patients with bleeding pseudoaneurysms (one post traumatic, one spontaneous) and one with a hemorrhagic pseudocyst of the pancreatic head were treated surgically with arterial suture and omentoplasty. Bleeding was controlled in all, without any postoperative mortality or morbidity. No rebleeding occurred with a follow up of 33, 26 and 12 months. Trancystic ligation of bleeding vessels with omentoplasty may be a useful approach, which should be compared to arterial embolization in the future.

KEY WORDS: Pancreas pseudoaneurysm pseudocyst hemorrhage surgery

Bleeding into a pancreatic pseudocyst is a rare and rapidly fatal complication requiring urgent treatment with arterial embolization[1] or surgical management[2]. Hemorrhage may be confirmed to the pseudocyst or, in the case of erosion of nearby organs, can appear in the gastrointestinal tract as hematemesis or melena. Mortality after emergency surgery is high, 33% in a recent report[3].

Pancreatectomy for distal lesions and arterial ligation for proximal lesions, which carry different complication and mortality rates[4], are the standard operative procedures. Surgical use of the greater omentum, utilized as a free or pedicled graft, is both protective and restorative[5]. We report our experience with bleeding pseudocysts of the pancreatic head treated with arterial suture followed by omentoplasty in 3 patients operated on during a 2 year period.

*Correspondance to:* Prof. Claude Huguet, Department of Surgery, Centre Hospitalier Princesse Grace, Avenue Pasteur, 98000 Monaco, Principauté de Monaco, Tel. 00-33-93.25.99.00

### PATIENTS

*Case 1* A 50 year-old male with a history of a motor boat accident 6 months previously, was admitted with epigastric pain and vomiting bile. At the time of the accident he had suffered· blunt abdominal trauma necessitating exploratory laparotomy for drainage of a pancreatic hematoma. One month later he was again operated on as on emergency on two occasions because of bleeding originating from the superior mesenteric and renal artery. Angiography demonstrated a small arterial false aneurysm from the pancreaticoduodenal artery in the proximity of the suprior mesenteric artery. Of interest was a thrombosis of the hepatic artery,the liver being perfused only by the superior mesenteric artery through the pancreaticoduodenal arteries. This finding contraindicated arterial embolization.

On admission, examination revealed epigastric tenderness without a palpable mass. Laboratory studies showed: hematocrit 37%, hemoglobin 12.6% g/L, amylase 236 IU/L. Emergency abdominal CT scan showed a fluid collection of the pancreatic head and, after injection of contrast, a large pseudoaneurysm was detected close to the superior mesenteric artery (Figure 1A).

On exploratory laparotomy a hard pulsatile mass in the head of the pancreas was confirmed. The aorta above the celiac trunk was clamped for 11 minutes and the aneurysm opened. There were two bleeding orifices that were sutured with 4–0 polypropylene (Prolene) stitches. The operation was ended by obliteration of the cavity with a pedicled graft of the great omentum fixed several 4–0 polyglactine stitches (Vicryl). The

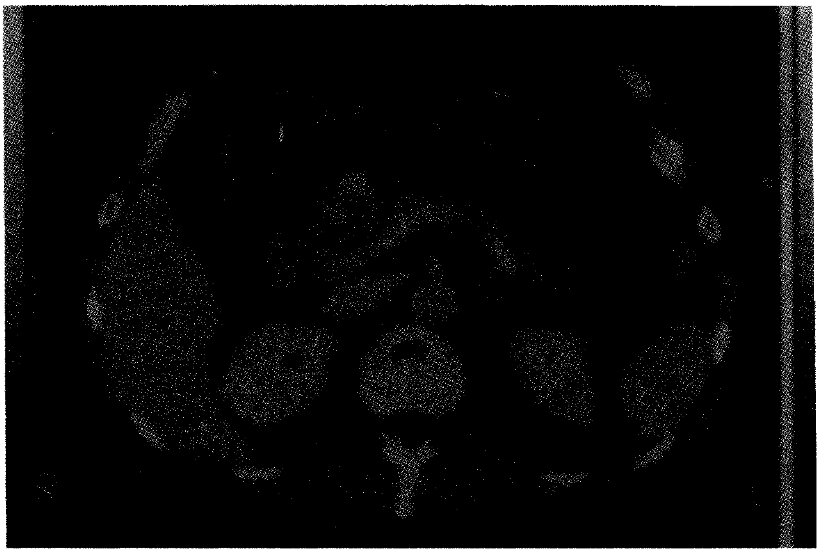

**Figure 1A** Preoperative abdominal CT scan, after injection of contrast media, showing a pseudocyst of the head of the pancreas with a large pseudoaneurysm close to the superior mesenteric artery.

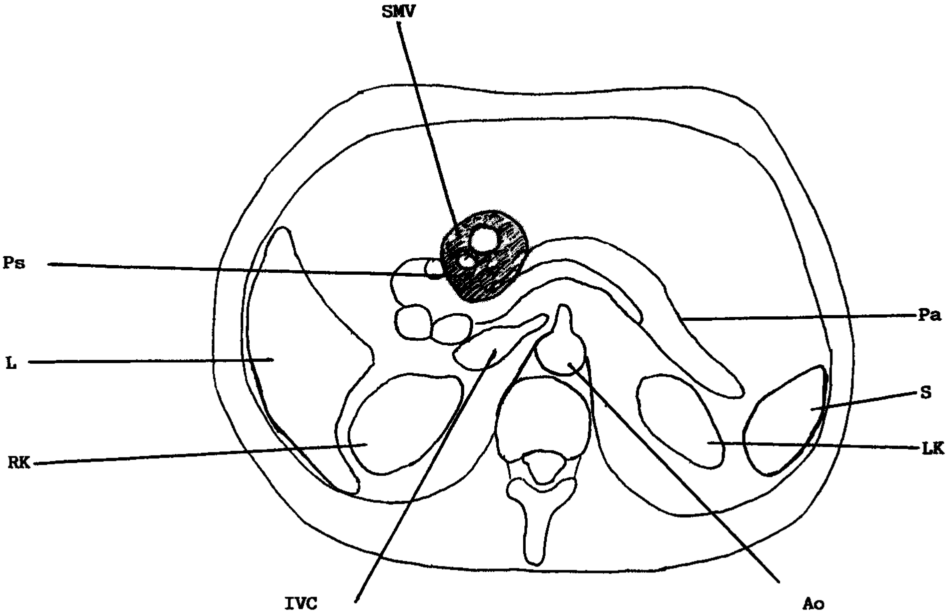

**Figure 1B** Explanatory diagram.: Spleen: L: Liver: Pa: Pancreas; Ao: Aorta: IVC: Inferior vena cava; RK: Right Kidney: LK: Left Kidney: Ps: Pseudoaneurysm: suppress: SMV: Superior Mesenteric Vessel.

patient recovered well and was discharged on the 12th postoperative day. The control CT scan demonstrated the disappearance of the pseudoaneurysm (Figure 1B). 33 months postoperatively the patient is doing well without any symptom of pancreatitis or pancreatic insuffiency. (Figure 1C).

*Case 2* A 79 year-old man began to suffer from anorexia followed by vomiting, during the postoperative course after prostatic resection for adenoma.

He denied alcoholic abuse. No abdominal mass was palpable. WBC was 17550 cells/$mm^3$, hematocrit 32%, hemoglobin 10.6 g/L, alkaline phosphate 548 IU/L and amylase 69 IU/L. An upper gastrointestinal contrast study showed extrinsic compression of the posterior wall of the stomach. The CT scan of the abdomen revealed a 6 cm lobulated pseudocyst of the pancreas filled with contrast media (Figure 2A).

Because his general condition was deteriorating the patient underwent emergency exploratory laparotomy. The pancreatic head was deformed by a nonpulsating round mass compressing the duodenum. The mass was opened and a large amount of blod clot was evacuated. There were two bleeding points which were sutured with 3–0 polyglactine stitches (Vicryl). The right part of the greater omentum was prepared as a pedicle graft and introduced and fixed inside the pseudocyst cavity. The patient left hospital on the 14th day postoperatively with a satisfactory control CT scan (Figure 2B). At the last follow up he is well 26 months after. (Figure 2C)

*Case 3* A 32-year-old man who was not alcoholic suffered from recurrent attacks of epigastric pain, he was treated with anti-H2 drugs which proved ineffective. Abdominal ultrasonogram revealed an "hepatic cyst" measuring 7 cm in diameter.

On admission physical examination was characterized by a right upper quadrant tender mass and laboratory values were in the normal range. An abdominal CT scan showed a normal liver but a lobulated pancreatic cyst, 7 cm in diameter.

The patient underwent exploratory surgery. In the head of the pancreas there was a non pulsatile mass extending laterally to the hepatic pedicle, inferiorly through the transverse mesocolon and displacing posteriorly the duodenum in the lesser sac. The mass was trapped and blood clots were aspirated. There were four independent cavities all of them hemorrhagic without any active bleeding.

Histologic examination did not show any malignancy. The pseudocyst was filled completely with an appendage of the great omentum. Postoperative course was uneventful, on the 10th postoperative day the patient was discharged and he is well 12 months postoperatively.

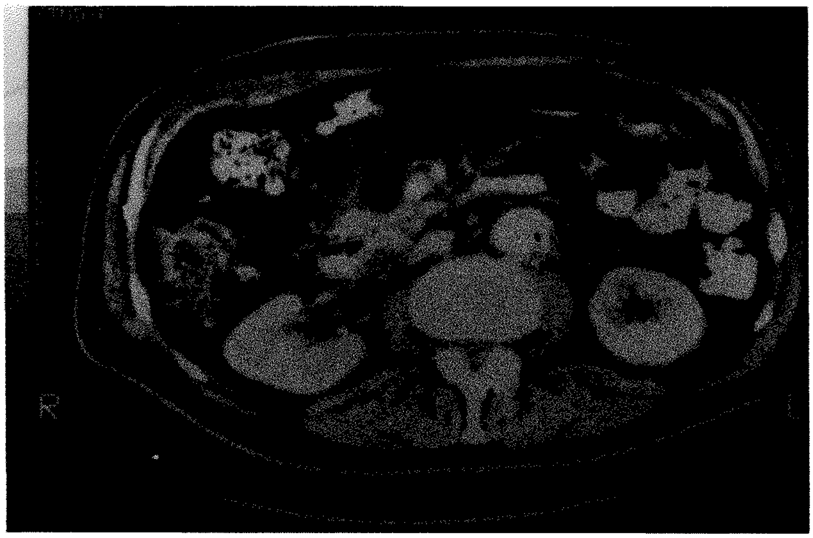

**Figure 1C** Abdominal CT scan on the 12th postoperative day showing the great omentum into pseudocyst of the pancreas with disappearance of the pseudoaneurysm.

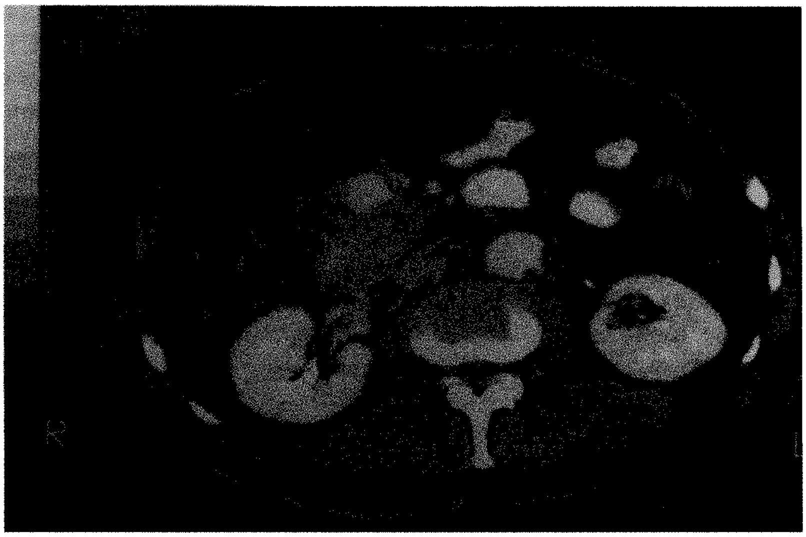

**Figure 2A** Preoperative abdominal CT scan showing a lobulated pseudocyst of the pancreatic head filling with contrst media.

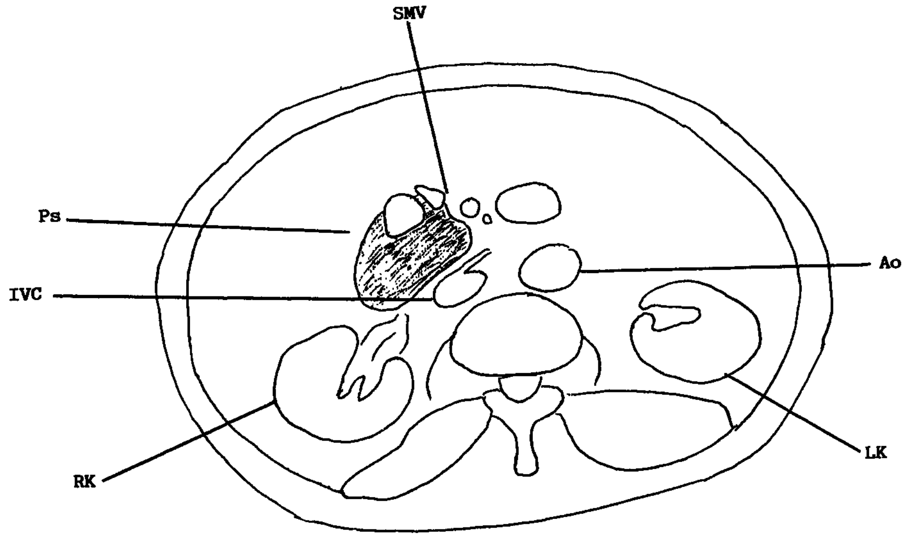

**Figure 2B** Explanatory diagram: suppress: Ao: Aorta: IVC: Inferior vena cava; RK: Right Kidney: LK: Left Kidney: Ps: Pseudoaneurysm: SMV: Superior Mesenteric Vessel.

### DISCUSSION

Patients with pancreatic pseudocyst have a 10% chance of bleeding into the gastrointestinal tract, into the peritoneum or into the pseudocyst itself[6,7]

Hemorrhage results from enzymatic erosion of parietal vessels and adjacent visceral organs in the presence of necrosis or sepsis. Bleeding is usually rapid and requires a prompt intervention, but when it is confined to the pancreas symptoms are vague a non-specific, leading to a delay in diagnosis.

Two of our patients were operated on in an emergency as their bleeding was active, while the third presented a symptoms which were, at first, underevaluated.

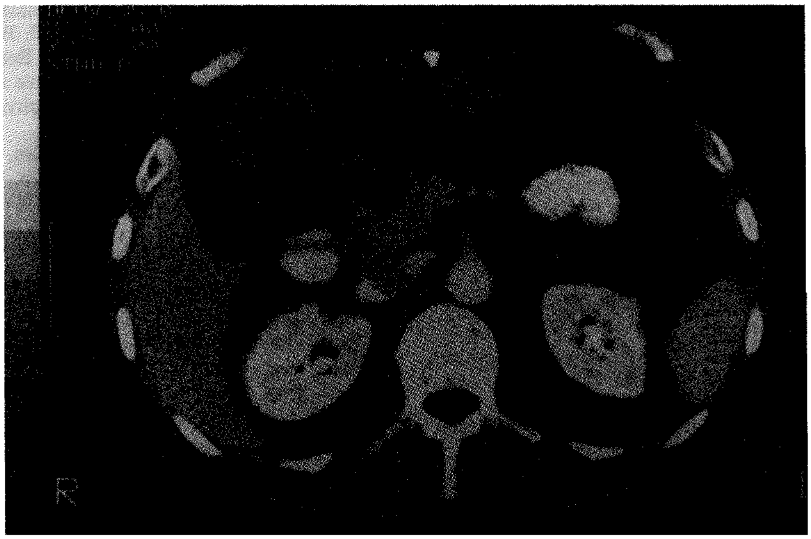

**Figure 2C** Abdominal CT scan on the 14th post operative day showing the great omentum into pseudocyst of the pancreas.

Diagnosis can be established with echo-doppler[8] and CT scan. Burke *et al.* reported the CT scan appearences of false aneurysms of the pancreas. Findings consisted of enhancing masses within or adjacent a non-enhancing pseudocyst[9].

Our patients were all studied by means of a CT scan which easily demonstrated the bleeding process. Doppler imaging which was not used in this series would certainly contribute to the diagnosis in the future. Angiography may be useful in determining the bleeding vessel and in providing embolization. Arterial embolization should be attemped whenever possible in stable patients. Huizinga successfully treated 4 patients with gelfoam embolization[1]. In a 10-year experience 19 patients with pancreatic arterial aneurysms were treated with embolization with 79% success rate[10]. Once hemostasis is achieved, large pseudocysts may require either surgical or percutaneous drainage, mainly to avoid the risk of infection[11].

The question is whether surgery for hemorhagic pancreatic pseudocyst is still indicated. Two recent reports recommended surgical treatment. In a series of 17 patients reported by El Hamel and co-workers, two thirds of the patients underwent pancreatic resection, the remaining had a transcystic arterial ligation with external or internal drainage of the pseudocyst[3]. Postoperative mortality was as high as 20% after pancreatic resection. Bresler's series of 10 patients was characterized by 10% mortality rate. Five patients have their bleeding vessel ligated and the psedocyst drained while the remaining patients and the pancreas resected with the same 20% mortality[2].

The three patients of the present series had the pseudocysts localized in the pancreatic head. Arterial suture was followed by omentoplasty without drainage.

The omentum is usually used intact by fixing one side of the extremity in the cystic cavity with interrupted sutures. In other instances, such as large cavities or in case of short omentum, a pedicled omental graft can be obtained.

The omental vascularity, composed of branches of the right and left gastro-epiploic arteries, has to be respected. Methods to increase the length of the omentum have been described by Das[12].

Pseudocysts and pseudoaneurysms are the result of enzymatic autodigestion of pancreatic parenchyma and vessel wall. The omentoplasty which obliterates the pseudocyst space may absorb pancreatic juice and/or blood. This could reduce the risk of fistula and rebleeding observed after transcystic ligature of bleeding vessels and external drainage[3,4]. One criterion for success of omentoplasty for pancreatic pseudocysts is the integrity of the main pancreatic ducts which could be assessed by preoperative endoscopic retrograde cholangio-pancreatography which was not used in our cases because of the emergency.

This conservative surgical approach of bleeding pseudoaneurysms and pseudocysts of the pancreatic head is to be recommended and preferred to Whipple resection which carries a high mortality rate under emergency conditions.

### REFERENCES

1. Huizinga, W. K. J., Kaliden, I. M., Bryer, J. V., Bell, P. S. H. and Baker, L. W. (1984) Control of major hemorrhage associated with pancereatic pseudocyst by transcatheter arterial embolization. *Br. J. Surg.*, **71**, 133–136.
2. Bresler, L., Boissel, P. and Grosdidier, J. (1991) Major hemorrhage from pseudocysts and pseudoaneurysms caused by chronic pancreatitis: surgical therapy. *World J. Surg.*, **15**, 649–653.
3. El Hamel, A., Parc, R., Adda, G., Bouteloup, P. Y., Huguet, C. and Malafosse, M. (1991) Bleeding pseudocyst and pseudoaneurysm in chronic pancreatitis. *Br. J. Surg.*, **78**, 1059–1063.
4. Stabile, B. E., Wilson, S. E. and Debas, H. T. (1983) Reduced mortality from pseudocyst and pseudoaneurysm caused by pancreatitis, *Arch. Surg.*, **118**, 45–51.
5. Samson, R. and Pasternak, B. M. (1979) Current status of surgery of the omentum, *Sur. Gynecol. Obstet.*, **149**, 437–442.
6. Fekete, F., Laignau, P. and Belghiti, J. (1980) Les hemorrhagies digestives au cours des pancreatites chroniques, *Gastrointestinal Clin. Biol.*, **4**, 551–555.
7. White, A. F., Baum, S. and Buranasiri, S. (1976) Aneurysms secondary to pancreatitis, *AJR*, **127**, 393–396.
8. Derchi, L. E., Biggi, E., Cicid, G. R., Bertoglio, C, and Neumaier, C. E. (1984) Aneurysms of the splenic artery: noninvasive diagnosis by pulsed Doppler sonography, *J. Ultrasound Med.*, **3**, 412–44.
9. Burke, J. W., Erickson, S. J., Kellum, C. D., Tagtmeyer, C. J., Williamson, B. R. J., and Hansen, M. F. (1986) Pseudoaneurysms complicating pancreatitis: detection by CT, *Radiology*, **161**, 447–450.
10. Mandel, S. R., Jaques, P. F., Sanofsky, S. and Mauro, M. A. (1987) Nonoperative management of peripancreatic arterial aneurysms, *Ann. Surg.*, **205**, 126–128.
11. VanSonnenberg, E., Wittich, G. R., Casola, G. *et al.* (1989) Percutaneous drainage of infected pseudicyst: experience in 101 cases, *Radiology*, **17**, 757–759.
12. Das, S. K. (1976) The size of the human omentum and the methods of lengthening it for transplantation, *Br. J. Plast. Surg.*, **29**, 170–174.

